# Academy of Stomatology

**Published:** 1895-01

**Authors:** George D. B. Darby

**Affiliations:** Secretary


### ACADEMY OF STOMATOLOGY.

Meeting held December 10, 1894.

### DISCUSSION OF DR. TRUMAN'S PAPER.

(For Dr. Truman's paper, see page 11.)

*Dr. Kirk.*—I am exceedingly gratified that Dr. Truman has investigated this subject so minutely. It is one in which I have been very much interested for some time.

In respect to coagulants, my own research was simply to decide the question whether they were self-limiting, or set up self-limited barriers to their further penetration, and I demonstrated to my own entire satisfaction that they were not self-limiting.

The phase of the matter which Dr. Truman has presented here to-night is one which I can hardly discuss. It will need a careful study of the paper, involving as it does the question of the relative diffusibility of these substances before it can be discussed intelligently.

There is a matter that I think Dr. Truman thoroughly recognizes, but it should be noted here, and that is, that the diffusibility of a coagulant substance may be varied, by differences in the strength of its solution, as by using different dilutions we get different effects: differences in density of coagulation and different rates of diffusion.

As to these two teeth respecting which Dr. Truman has asked my opinion, in one the inlet tube seems to be stopped up. I should say that the failure in this case to get the effect of the chloride of zinc through the cemental wall was due to a stop or break in the continuity of the column of zinc chloride solution. The tube appears to be plugged up with something.

I spent a great deal of time before I could get a perfectly solid column of liquid between the pulp-chamber and the inlet tube in the experiment of which this is the duplicate. I found, I suppose, in a half-dozen cases, or may be more, perhaps ten cases, in arranging the inlet tube in contact with the tooth, that I had great difficulty in getting a continuous column of liquid.

I know that the teeth Dr. Truman examined in my laboratory afforded, to my mind, conclusive proof that the zinc chloride had penetrated the cemental wall.

*Dr. Truman.*—So I thought at the time.

*Dr. Kirk.*—As to its being a question of a crack at the bifurcation of the root, I remember in one of my experiments coagulation took place at the root bifurcation, and I attributed that not to the supposition that there was an anatomical break here, but to the fact that the structure was thinner at that point, and the zinc chloride solution following the shortest course coagulation was started at that point. In the other cases it did not tend to start at the apex of the root, but near to the juncture of the enamel cap with the cementum. That is where coagulation occurred in the case that I reported as successful, which I exhibited in New York. Unless it was a break in the tooth-structure, it must have penetrated through the cemental wall, in my judgment.

I am quite interested to hear the views of Dr. Harlan on the subject. It is a very fortunate coincidence that he is with us this evening. We have been hearing so much on this one side of the question that I am sure it will be gratifying to all of us to hear the other side of the case stated.

*Dr. A. W. Harlan*, Chicago.—Mr. President and gentlemen, it is a very happy accident that brought me here this evening. I was entirely unaware that the subject was to be discussed, and I did not know that a paper was to be presented. I did not come forward at this time to state the other side of the question, because I am going to do that in New York, at the January meeting of the First District Dental Society, but I am going to make two or three queries which may have some bearing on this matter.

The first one is this: If zinc chloride is such a powerful penetrating agent, why is it that it does not preserve the portion of the pulp that is dead and left in a root that has been treated with zinc chloride and afterwards had a root-filling placed over it?

If carbolic acid is such a powerful penetrating agent in the apical portions of a dead pulp, why is it that an abscess inevitably follows after an incomplete root-filling, when that portion of the pulp has been thoroughly soaked and saturated as far as it may be with carbolic acid? If creosote, which is practically (or partially) soluble in water, is applied for weeks and months to these stump-ends of dead pulps, and afterwards a root-filling is introduced, whether it is metal or oxychloride of zinc, or gutta percha, or any other agent that will not force the portions of the dead pulp through the root, why is it we subsequently have an abscess if these are antiseptic as well as coagulating agents? Mr. President, it seems to me this is one of the questions that ought to be settled. I deny (without giving my reasons now) that zinc chloride or carbolic acid or creosote or bichloride of mercury—these are the four agents, I think, that have been mentioned here—are powerful penetrators of albuminous matter in living teeth.

I will give the reasons at the First District Dental Society of New York.

Again, the handling of pulpless teeth successfully by the men in this room has been largely brought about by the agitation of the question of the use of coagulating agents. Well, perhaps I am one of the guilty ones. I spent a great deal of time on it. I read the first paper on the subject in October, 1881, in Cincinnati, and from that time to this I have been engaged with matters in this connection.

I do not expect any one to answer the queries I have presented here to-night, because there is no man in the room who does not know that it is true that abscess almost invariably follows after the treatment of stump ends of dead pulps of teeth that are left there, and when the whole pulp has been soaked for weeks and months with creosote, and the tooth has afterwards been filled. Unless some change has taken place that will produce an encystment of the apex of the tooth, there will be an alveolar abscess there,—blind or with a fistula.

One of the reasons why a coagulating agent is not a fit substance to use in pulpless teeth is, that a coagulated body is the fittest food for micro-organisms. Dr. Truman smiles. Ernest LaPlace, of Philadelphia, I believe, is one of the authorities for that statement. W. D. Miller is also another authority. Hueppe is another. Koch is another. I do not need to mention others to the effect that a coagulated body is the fittest food for micro-organisms.

*Dr. J. Y. Crawford*, Nashville.—I have been very much entertained with the paper, as well as very much interested in the discussion. I would not presume to interject any crude and unprepared remarks. I have some views, it is true, but for fear they might mar the scientific trend of thought thus far expressed, I would prefer not to make them known.

*Dr. Gordon White*, Nashville.—I am entirely undecided on these subjects. I have made some little investigation, but nothing that has proved entirely satisfactory to my mind, as to which is the best agent to use. I find that in root-canals perfectly filled I have very little trouble with them afterwards.

I am, indeed, very glad to have this opportunity of listening to the paper and hearing the discussion from the gentlemen who have preceded me.

*Dr. C. R. Butler*, Cleveland.—I am, as several others have expressed themselves, unable to go into this discussion with any degree of intelligence. It has been presented in an altogether different manner from anything I have ever heard before. It seems to be based upon thorough investigation, and is, to a very large extent, original. It is a question that has been perplexing practitioners all along. It seems as though this all-important question of the treatment of pulp-canals or pulpless teeth was about to be settled, so that we would have some intelligent line of practice and mode of handling them, so that we would not be all at sea.

*Dr. C. S. Butler*, Buffalo.—I learned a week or ten days ago that Dr. Truman would present a paper upon this subject to-night, and I

thought I must, if possible, get to Philadelphia and hear the paper, which, to my mind, is the clearest presentation of that phase of the subject I have ever heard. I look forward now to the time when I can study it at my leisure and get a better comprehension of it.

Dr. M. L. Rhein, of New York, expressed his pleasure at having had the opportunity of hearing the paper, and believed, with the others, that it was a paper which required careful study at leisure, to enable one to determine the exact value which it unquestionably possesses. He thought, with one or two of the preceding speakers, that the points under consideration were nearer solution than they had ever been before in the history of our profession.

*Dr. Charles J. Essig.*—Mr. President and gentlemen, I did not intend to enter into the discussion, but I have been listening with a great deal of pleasure to Dr. Truman's paper. It occurred to me that the sense of the paper was not so much as to the value of certain agents in root-filling, or in the treatment of roots, as it was of the relative penetrating power of certain agents. And while I think his paper was a very valuable and thoughtful one and a good one in every respect, still I conceive that in order to determine a question of that kind it would be necessary to take up a series of the most careful experiments, and the nearer we could come to the condition of the mouth, the better able we would be to demonstrate the penetrating power of certain agents.

It would be necessary, it seems to me, to take pulpless teeth and fill the roots with an agent and subject them to the temperature of the mouth for a certain length of time, and then make microscopic sections of the tooth and examine them under high power, to determine the penetrating power of certain agents.

I do not understand Dr. Truman's paper as condemning coagulants in the treatment of pulpless teeth. As I understand it, the point of his paper was what the relative penetrating power of certain agents would be.

I should imagine that such a matter could be very accurately determined by a series of experiments with microscopic sections, but I cannot see how we can arrive at anything by a few experiments with glass capillary tubes, considering that they present different conditions entirely from those we meet in the mouth or teeth; and while it is of a great deal of value to observe these subjects even in the light in which they are here presented, it would be better still to first demonstrate beyond a doubt by very correct experiments, such as I have alluded to, which could be done if we could find the time and the enthusiasm, after the day's labor, to prosecute such work. It would be very difficult, as all such experiments are.

Now, while the specimens were going around, I could not help thinking of the experiments we used to make with amalgams, of filling glass tubes and then subjecting them to certain theoretical tests, and it was not until we brought them as near to the conditions of the mouth as possible that we began to learn something. When the experimental test fillings were subjected to the fluids of the mouth, at the temperature of the mouth, we found they behaved almost the same as they did in the mouth.

*Dr. A. H. Porter.*—I believe that in pathological treatment the more nearly it is identical with normal physiological action the more it is to be desired. In the formation of the tooth-substance from the blood-plasma nature has a certain purpose to effect. In any cell-proliferation where a dense, hard structure is to be produced, as in the odontoblastic formation, we have virtually a natural coagulation.

The main factor to be considered in an organ exposed to the impact of an external environment is its resistance to pressure. According to the strength and duration of this resistance is the vaso-motor mechanism called into play and the metabolic and circulatory activities regulated.

In the case of the visual and aural apparatus, these activities are at a maximum on account of the rapid, oscillating character of the ethereal and aërial environment forming light and sound.

In the case of tooth-structure, however, nature opposes a mechanical strain of comparatively long duration.

This permanent force is met by the permanent structure of enamel and dermal appendages generally where the circulatory activities are at a minimum.

The artificial coagulation demonstrated by the essayist approaches this condition, and is to be valued for its experimental character.

*Dr. Kirk.*—One point has occurred to me since Dr. Harlan spoke, and it seems to me his queries ought not to go by without some further consideration.

I believe it is undoubtedly true that coagulants form a suitable pabulum for the growth of certain micro-organisms, but what coagulants? Under what conditions? In an editorial in the *Dental Cosmos*, perhaps two years ago, I called attention to this same point, and it seems to me very important that when a coagulum is formed with any substance capable of producing coagulation when brought in contact with albumen or gelatin, we should recognize that we are producing a chemical compound by chemical reaction, and that it is a reaction which follows the law of all chemical combinations,—viz., it takes place in definite proportions by weight, that is, the relationship between the amount of coagulant required and the amount of albumen required to produce coagulation is exact and definite.

Now, if you produce a coagulum in an excess of albumen, you produce a compound which has no resulting excess of the coagulant. With, for example, zinc chloride, or bichloride of mercury, you can produce a coagulum that has no excess of the original zinc or mercury salt. Having the albumen in excess, all of the zinc chloride or sublimate has been used up in forming the coagulum, the compound is exactly balanced, and you thus have no doubt a coagulum which is, as it has in fact been demonstrated to be, a suitable pabulum for certain kinds of micro-organisms to grow upon. After you have formed your coagulum, however, if you provide an excess of the coagulant antiseptic, then you must certainly render the coagulum antiseptic, exactly the same as you would any other substance or tissue under the same circumstances. The bichloride of mercury or zinc chloride then being free to act, the question resolves itself into one of how long will this excess of antiseptic material remain active, and what is its antiseptic value.

Neutral coagula, in which there is no excess of the antiseptic which was used to form the coagulum, probably have little or no antiseptic value. Therefore, if you wish to get an antiseptic condition of a coagulum, you will have to use an excess of the coagulating substance.

The statement that Dr. Harlan made seemed to me to need that much qualification at least, to give us the right view of it. I hope the discussion of the subject will go on until we do know something more definitely about the proper position of these agents in the treatment of root-canals.

*Dr. James Truman.*—I had intended answering Dr. Harlan in the manner so ably illustrated by Dr. Kirk. We sometimes bow to authority without proper examination. I have never been able to make cultures on the class of coagulants described by Dr. Kirk, and I do not regard it as possible within a reasonable period. Again, I do not believe in Dr. Harlan's assertion in regard to the coagulating process, as he terms it, on the stumps of pulps. I do not believe that inevitably alveolar abscess follows that sort of treatment. On the other hand, I am well satisfied that it does not follow it. This is simply dogmatism, so is the other.

In reply to Dr. Essig's remark, that "this is not a carefully prepared paper——"

*Dr. Essig.*—No, not that; but that it did not go far enough.

*Dr. Truman.*—I admit that the paper is not exhaustive, but the experiments were, and if they were not, I do not know how they can be made so. I have spent three months on these, and have repeated them over and over again. It is true others may have been able to conduct them more successfully. I cannot conceive how it would be possible to make any essential difference by keeping the tubes at the temperature of the mouth. He asserts there was not sufficient care in microscopic experiments.

*Dr. Essig.*—I beg your pardon, I have not seen any microscopic specimens at all.

*Dr. Truman.*—My feeling was that the sections prepared in the course of these examinations would not be understood by all present. It was my ambition and desire to carry the stain with the coagulation into the tubes of the dentine, but I could not accomplish it. The coagulation would invariably leave the stain. All the stains with which I was familiar were tried, and I then consulted with the histologist of the department of medicine, University of Pennsylvania, but with no satisfactory result. Nitrate of silver will penetrate the tubuli without difficulty, but the stain is deep and so thorough as to render it almost valueless for observation. I have specimens at home and will be pleased to show them, but I did not think it worth while to bring them here to-night.

I have spent a large portion of my life in some forms of histological work, and I should know something about it; at all events, I do know how to differentiate normal tubes from those which contain coagulum. The whole character of the protoplasm is changed, and the tubes are so nearly obliterated that it becomes a matter of difficulty to trace the outlines of the original walls. My greatest desire is that this question should be settled, and I have tried to do something towards this end, and must believe that the work shown to-night carries it nearer a solution.

The subject was then passed.

### INCIDENTS OF OFFICE PRACTICE.

*Dr. Guilford.*—We are now on the eve of commemorating the discovery of anæsthesia, and it seems to me that we should not lose sight of the fact that there is always some danger connected with the anæsthetic state. It so happens that during last week some of our visitors who came from Buffalo brought with them an apparatus for producing forced respiration in cases of asphyxia, and exhibited it at one of the dental colleges in this city last Saturday. It excited so much interest that I took the liberty of asking Dr. Butler to exhibit it here to-night.

*Dr. C. S. Butler.*—It is only because I feel the importance of it to our profession, and perhaps because it may be new to some of you, that I have consented to come before you. It is simply an apparatus for use in cases where asphyxia has followed the administration of anæsthetics. It sometimes occurs that, notwithstanding all our methods of artificial respiration, we are unable to resuscitate the patient, or, rather, re-establish normal and natural respiration, and, consequently, the patient dies. It has been the opinion of the medical profession, on the other hand, that forced respiration should never be resorted to. Even as late as 1892, Dr. Marshall Hall, the celebrated English physician, stated in his "Hand-Book of Ready Reference," in cases of asphyxia, that forced respiration and the bellows should never be used. Three cases which have come under my personal observation satisfied me that the danger of forced respiration is entirely misconceived. It has been conclusively shown that forced respiration may be applied and the patient brought back to life again.

I will cite two cases of nitrous oxide asphyxia with which I am very familiar. One where the patient died; the other where the patient was saved. The first was that of a married lady below middle life, who came to a dentist in the city where I reside, and had nitrous oxide administered for the purpose of having a tooth extracted. She had taken the gas successfully on other occasions, but on this occasion, after the operation, it was observed that the patient had ceased to breathe. A messenger was despatched for a physician, and two physicians, who lived near by, came in a few moments, and made efforts to resuscitate the patient or to re-establish respiration, but without result, although the patient's heart continued to beat regularly and strongly for fifty-five minutes.

The other case occurred last July, in Buffalo. A lady, a trained nurse,—I mention that fact to show that she was of more than the average intelligence in regard to medical matters,—applied to one of my brother dentists for the administration of nitrous oxide for the extraction of a tooth. The nitrous oxide was given to the extent, perhaps to the amount, of six gallons, and the tooth was extracted; no unusual condition apparent during that time. For a moment or two after the tooth was extracted my friend noticed that she was not breathing. He endeavored to re-establish respiration by methods he had used on previous occasions, by opening the waist-bands and loosening the corsets, and endeavored by telescoping the short ribs suddenly, by placing his left arm beneath the patient's right arm, reaching over and letting go quickly. This failed of the desired result. He then despatched his assistant for me, and also, at the same time, to the nearest telephone to call the hospital ambulance, and with the request that they bring their forced respiration apparatus. I hastened at once to the patient with my friend Dr. Low, who was in the same building, and for about twenty minutes, or until the arrival of the ambulance, we worked by placing her on the floor, with shoulders elevated, by the Sylvester method of artificial respiration. It was probably twenty-five or possibly thirty minutes before the arrival of the hospital surgeon, and the apparatus for forced respiration was applied. In possibly twenty-five or thirty minutes after the forced respiration had been commenced the patient was able to be moved to the hospital, and in two hours after arriving at the hospital she was able to go about her usual duties. Now, there is not the slightest doubt in my mind that without the forced respiration apparatus she would never again have breathed normally, and would have died. Indeed, she came near dying after the surgeon arrived, her heart having nearly ceased to beat.

Another case of narcotic asphyxia from narcotic poisoning probably illustrates the value of the apparatus better than any other. It was the case of a young physician recently graduated, and who had just entered upon his professional career. Through an unusual number of coincidences he had been deprived of the normal quantity of rest and sleep for a period of five days and nights, and in order to sustain himself and continue his duties he had begun the taking of strychnine as a stimulant to brace him up. On arriving at his office about half-past one at night, he went into the bath-room and injected into his arm one-fortieth of a grain of strychnine; feeling no effect, two or three minutes afterwards he injected two-fortieths, and in a moment later another fortieth; beginning to feel some tremor, but having no apprehension of danger, to counteract it he began to use morphine hypodermically. This he continued as long as he was conscious. He was found in this condition at seven o'clock in the morning in the bath-room by his father. Physicians were despatched for at once, and at half-past seven a physician arrived with the forced respiration apparatus.

It was immediately seen that the case was a serious one, and they performed tracheotomy in order to facilitate enforced respiration, and for three days and nights continuously, with cessations a few moments at a time, the physicians and nurses continued to breathe for their young physician. During the seventy-two hours there was probably not more than fifteen minutes during the whole time in which the patient was physically able to breathe by himself, although after a few hours on the third day he was thoroughly conscious of all that was being done for him, and after a few hours spoke in a low whisper, but was still unable to breathe by himself. At the end of the seventy-two hours he was able to breathe quite comfortably, and very soon regained normal vigor, and in a few days went about his professional duties.

These three cases, it seems to me, establish very clearly the false position in which the medical world has held forced respiration. Since gentlemen in our city began to use the forced respiration method there have been saved by it over fifty persons who, humanly speaking, without forced respiration would have died.

A demonstration of the apparatus and its method of use was then given by the speaker.

Dr. Kirk, on behalf of Dr. Essig, presented to the museum some specimens of abnormal teeth, consisting of two pairs of teeth with roots fused together. A specimen also, of an upper cuspid having two roots; another, a pathological specimen of which there is no description. Also a preparation of lithium bitartrate, sent from London. Dr. Kirk said in regard to it,—

"I wrote a communication some time ago on the lithium bitartrate as a remedy in lithæmia, and especially the cases of lithæmia which result in certain forms of pyorrhœa alveolaris. The preparation is made by Alfred Bishop, of London, in the granular effervescent form. It was suggested to him by reading a paper, to which I have referred, in the *Lancet.* I suspect, although I do not know, that it contains something besides lithium bitartrate. It is marked as containing five grains of lithium bitartrate to a drachm of the powder or granules, and I presume that the balance of fifty-five grains of the material is possibly bicarbonate of sodium or potassium, to bring about the effervescent quality.

"I have another preparation here, made by McKessin & Robins, of New York, which they call tartarlithine. It contains nothing in the shape of other alkaline salts, and is effervescent. It has added to it a small amount of saccharin to modify the sharp acid taste.

This preparation is the one I have been using most successfully in my own work.

"I would like to say that if there are any gentlemen present sufficiently interested to observe cases under treatment of this particular lithium salt, and I find it has a value considerably above any of the lithium preparations I have ever tested, I will be very glad to furnish him with the necessary material to do it."

*Dr. Deane.*—We have from time to time in our Philadelphia Society heard of English teeth and of their fine texture. We have in the Pennsylvania Dental College a young Englishman who brought some of Ash's teeth with him, and asked permission to put them on a plate,—bridge-work. I borrowed it this evening to show it to the members of the society. It may prove to be interesting to some. The teeth are set on a pin, and cemented by melted sulphur. He has left the first bicuspid loose so it can be taken off. The teeth seem to be as fine on the inner portion where the tooth is ground out as they are on the surfaces. He tells me they have no trouble in splitting or breaking when they are fastened on the plate by the sulphur. The bridge is made of German silver, and not intended for fine work other than the ordinary college work of the junior year.

Adjourned.

George D. B. Darby,
*Secretary.*

